# Caracterización molecular de las variantes del virus de Epstein-Barr detectadas en la cavidad oral de adolescentes de Cali, Colombia

**DOI:** 10.7705/biomedica.4917

**Published:** 2020-08-20

**Authors:** Daniela Arturo-Terranova, Sebastián Giraldo-Ocampo, Andrés Castillo

**Affiliations:** 1 Departamento de Biología, Facultad de Ciencias Naturales y Exactas, Universidad del Valle, Cali, Colombia Universidad del Valle Departamento de Biología Facultad de Ciencias Naturales y Exactas Universidad del Valle Cali Colombia; 2 Posgrado en Ciencias Biomédicas, Escuela de Ciencias Básicas, Facultad de Salud, Universidad del Valle, Cali, Colombia Universidad del Valle Posgrado en Ciencias Biomédicas, Escuela de Ciencias Básicas Facultad de Salud Universidad del Valle Cali Colombia

**Keywords:** infecciones por virus de Epstein-Barr, boca, filogenia, adolescente, Colombia, Epstein-Barr virus infections, mouth, phylogeny, adolescent, Colombia

## Abstract

**Introducción.:**

El virus de Epstein-Barr (EBV) es un virus ubicuo y oncogénico, asociado con el desarrollo de enfermedades como la mononucleosis infecciosa, el linfoma de Burkitt, el carcinoma nasofaríngeo y otras neoplasias. Actualmente, se reconocen dos subtipos: EBV-1 y EBV- 2, que tienen diferencias genéticas con sus antígenos nucleares (*Epstein-Barr Nuclear Antigens*, EBNA). Debido a la gran heterogeneidad y variabilidad encontradas en la proteína LMP1 del virus, se han descrito variantes asociadas con ciertas enfermedades o con regiones geográficas específicas.

**Objetivo.:**

Identificar y caracterizar molecularmente las variantes del EBV detectadas en la cavidad oral de 84 adolescentes de Cali, Colombia.

**Materiales y métodos.:**

Se hizo la amplificación por reacción en cadena de la polimerasa (*Polymerase Chain Reaction*, PCR) convencional, así como la purificación y la secuenciación del gen *EBNA3C* se realizó para subtipificar el virus y del dominio C-ter de la proteína LMP1 para identificar variantes. Además, se llevó a cabo un análisis filogenético y de variantes nucleotídicas de las secuencias obtenidas comparadas con variantes patogénicas y geográficas reportadas en el GenBank (*National Center for Biotechnology Information,* NCBI).

**Resultados.:**

El subtipo viral predominante fue el EBV-1 (79 %); el 72,6 % se agrupó con la variante patogénica Raji, derivada de linfocitos B de un paciente con linfoma de Burkitt; el 13,7 % se relacionó con una variante de origen geográfico del Mediterráneo y otro 13,7 % no se agrupó con ninguna de las variantes de referencia.

**Conclusiones.:**

Este es el primer estudio que reporta variantes del gen *LMP1-EBV* en Cali, Colombia. Se requieren nuevos estudios para caracterizar la variante sin identificar y determinar si es patogénica o si es una variante geográfica presente exclusivamente en la ciudad.

## Abstract (en)

**Introduction:**

The Epstein-Barr virus (EBV) is an ubiquitous and oncogenic virus associated with the development of diseases such as infectious mononucleosis, Burkitt’s lymphoma, nasopharyngeal carcinoma, and other neoplasms. Currently, two types are recognized: EBV-1 and EBV-2, which have genetic differences with their EBNA nuclear antigens. Likewise, due to the high degree of heterogeneity and variability found in the LMP1 protein of the virus, variants associated with pathogenesis or specific geographic regions have been described.

**Objective:**

To identify and characterize molecularly EBV variants detected in the oral cavity of 84 adolescents in Cali, Colombia.

**Materials and methods:**

Conventional PCR amplification, purification, and sequencing of the gen *EBNA3C* were carried out to typify the virus and the C-ter domain of the LMP1 protein to identify variants. We also conducted a phylogenetic and nucleotide variant analysis of the obtained sequences *versus* pathogenic or geographic variants reported in GenBank-NCBI.

**Results:**

The predominant viral subtype was EBV-1 (79%); 72.6% was grouped with the pathogenic variant Raji, derived from B lymphocytes of a patient with Burkitt›s lymphoma, 13.7% was related to a variant of Mediterranean origin, and 13.7% was not grouped with any of the reference variants.

**Conclusions:**

This is the first time that variants of LMP1-EBV have been identified in Cali, Colombia. Additional studies are necessary to characterize the unidentified variant and to determine if it is pathogenic or if it is just an isolate present in the city of Cali.

El virus de Epstein-Barr fue descrito por primera vez en 1964 por Epstein, Achong y Barr ^1^. Pertenece a la familia Herpesviridae, a la subfamilia de Gammaherpesvirinae, y es el prototipo del género *Lymphocryptovirus*^2^.

El vehículo de trasmisión del EBV es principalmente la saliva ^3^, aunque se ha detectado ADN del virus en la leche materna y en algunas secreciones genitales, pero la información sobre esto último es todavía poca ^4^.

La partícula viral del EBV presenta una envoltura que contiene en su interior una nucleocápside conformada por un genoma de ADN de doble cadena de 172 kpb rodeada de una cápside icosaédrica compuesta por 162 capsómeros ^5^. Se han descrito dos subtipos virales del EBV, el EBV de tipo 1 (EBV-1) y el de tipo 2 (EBV-2). Esta clasificación se basa en la presencia de polimorfismos en los genes que codifican los antígenos nucleares EBNA3A, EBNA3B y EBNA3C ^6^. Sin embargo, dado que estos polimorfismos no son suficientes para describir toda la variación natural del EBV, también se han descrito variantes con base en polimorfismos encontrados en otros antígenos virales, como la proteína latente de membrana 1 (LMP1) ^7^.

Clínicamente, el EBV se caracteriza por infectar persistentemente al 90 % de la población mundial ^8^; además, es el agente etiológico de la mononucleosis infecciosa en niños ^9^ y adolescentes ^10^, y de la enfermedad linfoproliferativa posterio a trasplante ^11^. Según la *International Agency for Research on Cancer,* (IARC*)*, se clasifica como un carcinógeno del grupo 1 ^12^^,^^13^ asociado con tumores como el linfoma de Burkitt, el de Hodking y el no Hodking ^14^ y el carcinoma nasofaríngeo ^15^. Aún no está totalmente dilucidada la etiopatogenia del EBV para el desarrollo de neoplasias malignas; sin embargo, en algunos estudios se ha propuesto que ciertas proteínas virales, como la proteína LMP1, estarían implicadas en dichas condiciones ^16^.

La proteína LMP1, codificada por el gen *BNLF1* viral, tiene un peso molecular de 66 kDa; cuando se expresa, se localiza en la membrana plasmática de las células infectadas y desempeña un papel importante en el desarrollo de las neoplasias ^17^. Está conformada por 386 aminoácidos sectorizados en tres dominios: un dominio N-terminal corto de los aminoácidos 1 a 23; seis segmentos hidrofóbicos transmembranales de los aminoácidos 24 a 186, y, por último, un dominio carboxilo terminal (C-ter) de los aminoácidos 187 a 386 ^18^. El dominio C-ter interactúa con proteínas celulares mediante regiones de activación denominadas CTAR1 y CTAR2, las cuales activan varias vías de señalización celular de la célula infectada ^19^.

Esta oncoproteína viral puede autorregularse por diferentes vías celulares ^20^ y posee la capacidad de transformar *in vitro* fibroblastos de roedores ^21^, y convertirlos en tumorogénicos *in vivo*^22^. Además, puede actuar como el receptor celular CD40 constitutivamente activado, perteneciente a la familia de receptores del factor de necrosis tumoral ^23^, para estimular múltiples vías de señalización, de manera independiente del ligando, lo cual promueve el crecimiento, la proliferación celular, la supervivencia y la inhibición de la apoptosis ^24^.

Dado que las secuencias de los ácidos nucleicos del *BNLF1* viral que codifican la región del dominio C-ter de la proteína LMP1 presentan una gran variabilidad entre los aislamientos con presencia del EBV, varios investigadores han propuesto los análisis filogenéticos de esta región para determinar subtipos o variantes moleculares según su origen geográfico y su asociación patológica ^25^^,^^26^.

Entre las variantes geográficas descritas, se han identificado las Med81, Med+ y Med- de la región del Mediterráneo; las China 1 y 2 de China; la NC de Carolina del Norte, y la Alaskan de Alaska. Entre las variantes identificadas en líneas celulares patogénicas, se cuentan: la Raji, la AG876 y la MUTU de pacientes africanos con linfoma de Burkitt ^27^^-^^29^; las cepas GD1, GD2, Cao y AKATA de pacientes con este linfoma o con carcinoma nasofaríngeo ^30^, y la B95.8 de un paciente con mononucleosis infecciosa ^26^.

En este sentido, las herramientas filogenéticas permiten describir espacial y temporalmente los virus con relación a su huésped, localizar el origen de una enfermedad en particular, determinar qué cepas y variantes poseen mayor posibilidad de propagación en el futuro, y determinar la vulnerabilidad de la población frente a un virus. El reconocimiento de las variantes y su posible asociación con regiones geográficas o diferentes enfermedades, podría contribuir al entendimiento de la enfermedad en la población.

En Colombia, los pocos estudios sobre el EBV se han centrado únicamente en determinar la seroprevalencia del virus y su relación con diversas enfermedades, por lo que hay muy poca información sobre sus variantes y su efecto en las personas. Por ello, el objetivo de este trabajo fue identificar y caracterizar molecularmente las variantes del virus de Epstein- Barr detectado en la cavidad oral de adolescentes de Cali.

## Materiales y métodos

### Tipo de muestra

Se analizaron 84 muestras de ADN positivas para EBV, procedentes de un estudio previo ^31^ aprobado por el Comité de Ética Humana de la Universidad del Valle (Acta N° 008-017). En el estudio citado, se tomaron 374 muestras de enjuague bucal de la cavidad oral de jóvenes adolescentes de Cali. El ADN se extrajo con el estuche PrepMan Ultra Sample Preparation Reagent™ de Applied Biosystems y se almacenó a -20 °C en agua libre de nucleasas hasta el análisis para detectar el EBV. Para la detección del ADN viral, se usaron los cebadores EBV-F; 5’-CCT GGT CAT CCT TTG CCA-3’ y EBV-R; 5’-TGC TTC GTT ATA GCC GTA GT-3’ y la técnica de PCR convencional. La reacción amplificó un fragmento de 95 pb y se logró un porcentaje de detección del 26 % (96/374).

### Determinación de subtipos del EBV

Para determinar la presencia de subtipos del EBV, se amplificó el gen viral *EBNA3C* con la pareja de cebadores propuesta por Kingman, *et al.*^32^, con los cuales el tamaño del amplicón producto de la PCR varía según el subtipo. Para el subtipo EBV-1, se obtiene un tamaño de 153 pb y, para el EBV-2, uno de 246 bp.

Los cebadores utilizados en la reacción de amplificación fueron: EBNA3Cfwd 5'- AGA AGG GGA GCG TGT GTT GT-3' y EBNA3Crev 5'-GGC TCG TTT TTG ACG TCG GC-3'.

Las siguientes fueron las condiciones de la PCR: una desnaturalización de cinco minutos a 95 °C; 35 ciclos de amplificación durante 30 segundos a 94 °C, 30 segundos a 60 °C y un minuto a 72 °C, y una extensión final de cinco minutos a 72 °C. El volumen y concentración de los reactivos fue: 4 µl de solución tampón de amplificación 5X (Tris-HCl 10 mM, pH 8,4, en solución tampón de KCl 50 mM), 0,8 µl de MgCl_2_ a 50 mM, 0,6 µl de cada cebador a 10 µM; 1,2 µl de dNTP a 5 mM y 0,2 µl de Taq polimerasa 5U. La concentración final de los reactivos fue: solución tampón 1X, MgCl_2_ 2 mM, 0,3 µM y 1U cada cebador, respectivamente. Se completó hasta un volumen final de 20 µl con 11,6 µl de agua libre de DNasas y 1 µl de ADN.

Como controles positivos, se utilizaron muestras anteriormente determinadas por PCR para cada uno de los subtipos y una positiva para infección viral simultánea; como control negativo, se empleó la mezcla de PCR sin añadir ADN.

Los productos de la amplificación se separaron por electroforesis en geles de agarosa al 1,5 % teñidos con bromuro de etidio y se visualizaron en un transiluminador de luz ultravioleta.

### Amplificación y secuenciación del gen BNLF1 del EBV

Para determinar la presencia de las variantes del EBV, se amplificó y secuenció un fragmento de 576 pb del dominio carboxilo terminal del gen viral *BNLF1* con la pareja de cebadores propuesta por Zuo, *et al*. ^33^^), (^^33^. Las variantes fueron analizadas filogenéticamente y caracterizadas molecularmente.

Los cebadores utilizados en la reacción fueron: BNLF1fwd 5'-GTG CGC CTA GGT TTT GAG AG-3' y BNLF1rev 5'-TTC CTT CTC TAA CGC ACT TTC TC -3'.

Las siguientes fueron las condiciones de la PCR: una desnaturalización de cinco minutos a 95 °C; 40 ciclos de amplificación de 45 segundos a 94 °C, de 45 segundos a 58 °C y de un minuto a 72 °C, y una extensión final de 10 minutos a 72 °C. El volumen y concentración de los reactivos fue: 4 µl de solución tampón de amplificación 5X (Tris-HCl 10 mM, pH 8,4 en solución tampón de KCl 50 mM), 0,28 µl de MgCl_2_ 50 mM, 0,8 µl de cada cebador 10 µM; 1,2 µl de dNTPs 5 mM y 0,2 µl de Taq polimerasa 5U. La concentración final de los reactivos fue: solución tampón 1X, MgCl_2_ 0,7 mM, de cada cebador 0,4 µM, DNTPs 0,3 mM y 1U de Taq. Se completó hasta un volumen final de 20 µl con 11,72 µl de agua libre de DNasas y 1 µl de ADN.

Los productos de la amplificación se secuenciaron directamente mediante el método de Sanger de la empresa Macrogen, Inc., Corea.

### Análisis filogenético

Para la construcción del árbol filogenético, se empleó el programa de análisis genético de evolución molecular MEGA, versión 7 ^34^, para analizar las secuencias obtenidas de nucleótidos de los fragmentos amplificados de la región carboxilo terminal del gen *BNLF1*, las cuales se compararon con secuencias de referencia de las variantes del EBV almacenadas en la base de datos del GenBank-NCBI con los números de acceso: V01555.2 (B95.8); AY961628.3 (GD1); HQ020558.1 (GD2); KC207813.1 (Akata); X58140.1 (Cao); KF717093.1 (Raji); DQ279927.1 (AG876); AY337723.1 (China 1); AY337724.1 (China 2); AY337722.2 (Med +); AY337721.2 (Med-); AY337726.2 (NC); AY337725.1 (Alaskan); y KF373730.1 (M81).

En el análisis, todas las secuencias de nucleótidos se alinearon con el algoritmo Clustal W ^35^. Posteriormente, utilizando el criterio de inferencia bayesiana y con el paquete Models se seleccionó el modelo evolutivo que mejor se ajustaba a los resultados obtenidos en el alineamiento de las secuencias. Por último, se evaluó el árbol filogenético con el algoritmo de máxima verosimilitud y 100 réplicas de remuestreo.

### Análisis de variantes

Los cambios de aminoácidos del dominio carboxilo terminal de la proteína LMP1, a partir de las mutaciones no sinónimas del gen *BNLF1*, se determinaron con el programa Blastn, utilizando la opción ‘CDS feature’ y, como referencia de comparación, la secuencia del EBV (número de acceso en el GenBank-NCBI: NC_007605).

## Resultados

### Determinación de los subtipos del EBV

**Figura 1:**
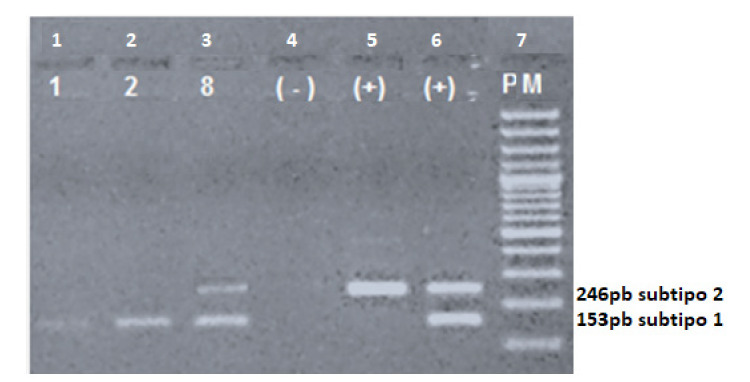
Tipificación del EBV mediante la amplificación del gen *EBNA3C* por PCR convencional. El subtipo 1 del EBV se caracteriza por presentar un tamaño de fragmento de 153 pb y, el subtipo 2, uno de 246 pb. En los carriles 1 y 2 se observa el EBV del subtipo 1, muestras 1 y 2; en el carril 3, la exposición simultánea a los dos subtipos, muestra 8; en el carril 4, el control negativo; en los carriles 5 y 6, los controles positivos del subtipo 2 y ambos subtipos, respectivamente; y en el carril 7, el peso molecular (PM).

De las 96 muestras positivas para el EBV, en 84 se logró amplificar el tamaño de los fragmentos para determinar los subtipos, y se obtuvo el subtipo EBV-1 en el 79 % (66/84) de estas muestras y, el subtipo EBV-2 en el 19 % (16/84), en tanto que dos de las muestras presentaron ambos subtipos (figura 1 y cuadro 1).

**Cuadro 1:** Relación de los aislamientos de adolescentes de Cali, Colombia, usados para identificar variantes con sus respectivas características

ID de la muestra	Cambios en los aminoácidos en la región C-terminal de la proteína LMP1*	Grupo de Variante	Característica de la variante
Cali EBV 1	A210N, G212S, H213N	Raji	Patogénica
Cali EBV 2	G212S, H213N	Mediterráneo	No patogénica
Cali EBV 3	G212S, N218K, G230E	Cali	Indeterminada
Cali EBV 4	G212S, H213N	Raji	Patogénica
Cali EBV 5	D210AN, G212S, V228E	Mediterráneo	No patogénica
Cali EBV 6	G212S, N218K, L226H	Raji	Patogénica
Cali EBV 7	G212S, H213N, S229T	Mediterráneo	No patogénica
Cali EBV 8	G212S, S229T	Mediterráneo	No patogénico
Cali EBV 9	G212S, H213N, L226H	Raji	Patogénica
Cali EBV 10	N218K, L226H, V228E	Raji	Patogénica
Cali EBV 11	G212S, L226H	Raji	Patogénica
Cali EBV 12	D210N, G212S, L248Q	Cali	Indeterminada
Cali EBV 13	L226H, G230E	Raji	Patogénica
Cali EBV 14	G212S, H213N, L226H	Raji	Patogénica
Cali EBV 15	G12S, S229T, L248Q	Cali	Indeterminada
Cali EBV 16	D210NA, G212S, H213N	Raji	Patogénica
Cali EBV 17	A210D, H213N	Raji	Patogénica
Cali EBV 18	G212S, L226H	Raji	Patogénica
Cali EBV 19	G212S, H213N	Raji	Patogénica
Cali EBV 20	G212S, L226H	Raji	Patogénica
Cali EBV 21	G212S, H213N	Raji	Patogénica
Cali EBV 22	G212S, H213N	Raji	Patogénica
Cali EBV 23	G212S, H213N, N218K	Raji	Patogénica
Cali EBV 24	G212S, H213N	Raji	Patogénica
Cali EBV 25	Al10D, G212S, H213N, G212S, H213N	Raji	Patogénica
Cali EBV 26	A210D, G212S, H213N	Raji	Patogénica
Cali EBV 27	G212S, S229T	Cali	Indeterminada
Cali EBV 28	D210N, G212S, H213N	Raji	Patogénica
Cali EBV 29	G212S, H213N,	Raji	Patogénica

*El cambio de aminoácido fue determinado por comparación con la secuencia de referencia ID: NC_007605

### Determinación de las variantes de EBV

**Figura 2:**
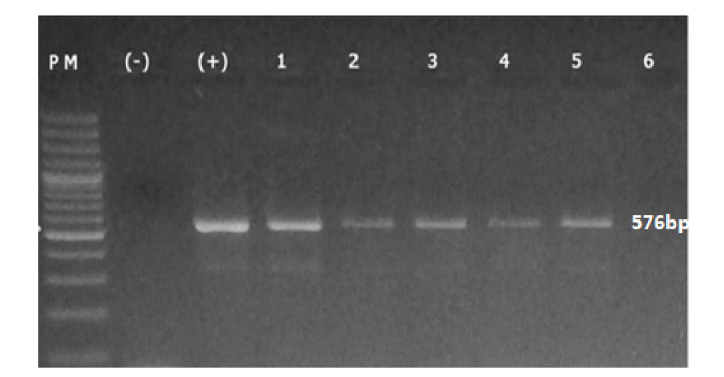
Amplificación por PCR convencional del fragmento C-terminal del gen *BNLF1* del EBV. En la figura se observa la amplificación de un fragmento de 576 pb que codifica para el dominio C-terminal de la proteína viral LMP1. PM: peso molecular; (-) control negativo; (+) control positivo; muestras 1 a 6

En cuanto a la determinación de las variantes del EBV, en 29 de las 96 muestras se logró amplificar y secuenciar un fragmento de 576 pb del gen *BNLF1,* correspondiente a la región que codifica el dominio C-ter de la proteína LMP1 viral (figura 2).

### Análisis filogenético e identificación de variantes

Las variantes de la proteína LMP1 se caracterizaron mediante la reconstrucción filogenética con secuencias prototipo del GenBank. El modelo de evolución para la elaboración de los tres análisis se determinó utilizando el programa MEGA 7, y se halló que el Jukes-Cantor (JC+G) era el más adecuado para el estudio; las tasas de sustitución encontradas para las secuencias fueron [AC]=[CG]=0,166; [AG]=0,083; [AT]=0,083, y [CT]=[GT]=0,166, y las frecuencias de bases, A=0,250; C=0,250; G=0,250; T=0,250, y p-inv=0,5.

**Figura 3:**
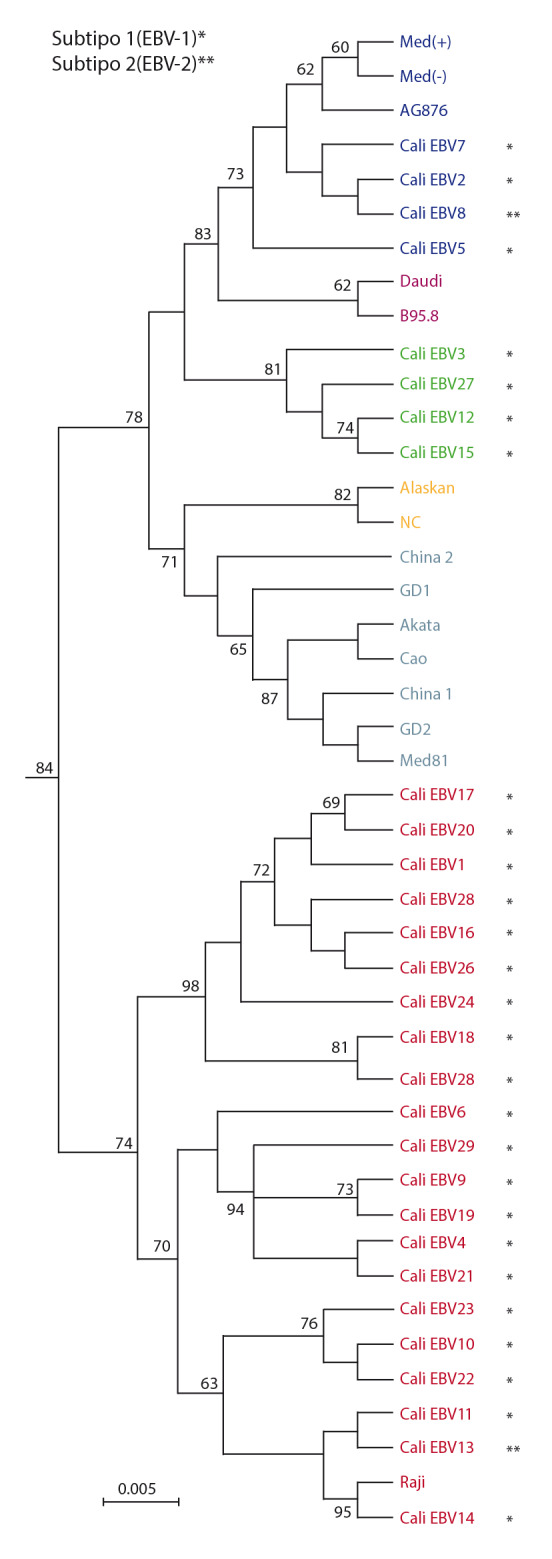
Filogenia de los EBV presentes en Cali, Colombia. Para la construcción del árbol filogenético se amplificó y secuenció un fragmento de 576 pb de la región C-terminal del gen *BNLF1.* En la figura se observan las agrupaciones filogenéticas obtenidas y valores de remuestreo mayores de 60 % obtenidos al realizar 1.000 réplicas. La escala indica el número de sustituciones por sitio y a cada variante se le asignó un color: Mediterráneo, azul; Raji, rojo; Cali, verde; Alaska y Carolina del Norte, amarillo; Daudi y B95.8, violeta.

En la figura 3 se muestra la distribución de las secuencias de los EBV detectados en Cali, en relación con las variantes de referencia del virus ligadas a un área geográfica o a alguna de sus manifestaciones clínicas.

Las 29 secuencias de los EBV detectados conformaron tres agrupaciones, una con los 21 aislamientos de Cali (72,6 %) relacionados con la línea celular Raji (KF717093.1, identidad del 99 %) con un remuestreo de 74 %; la otra agrupación presentó cuatro aislamientos de Cali (13,7 %) relacionados con el Mediterráneo (AY337722.2, identidad del 98 %) con un remuestreo de 83 % y, por último, cuatro muestras de Cali (13,7 %), con un remuestreo de 81 %, que no se agruparon con ninguna de las líneas celulares ni con las regiones geográficas consideradas en el análisis, por lo que se decidió denominarla como variante Cali (EBV Cali).

### Análisis de las variantes moleculares

Al analizar las variantes encontradas en los aislamientos de Cali en relación con el genoma de referencia del EBV, se encontraron 20 mutaciones no sinónimas; la más frecuente fue la Gly212Ser, en 86,20 % de los aislamientos, seguida de la His213Asn, con 58 %, y la Leu226His, con 27 % (cuadro 1).

**Figura 4:**
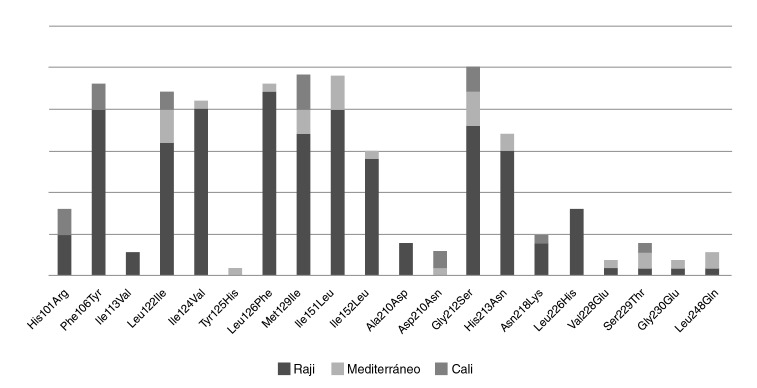
Distribución de las variantes moleculares identificadas en la región C-terminal de la proteína LMP1 de los EBV presentes en Cali. Se identificaron 20 mutaciones no sinónimas; la más frecuente fue el cambio de glicina por serina en la posición 212 (Gly212Ser), con un porcentaje del 86,2 %, seguido del cambio de metionina por leucina en la posición 129 e isoleucina por leucina en la posición 151, con un porcentaje de 79 %. En las muestras agrupadas con la variante patogénica Raji, se encontró un mayor número de cambios de aminoácidos, seguidas de aquellas agrupadas con las variantes Mediterráneo y Cali; los cambios de aminoácidos de alanina por ácido aspártico en la posición 210 (Ala210Asp) y de leucina por histidina en la posición 226 (Leu226His), se identificaron exclusivamente en los aislamientos relacionados con la variante Raji, en tanto que el cambio de tirosina por histidina en la posición 125 (Tyr125His) se identificó exclusivamente en los relacionados con la Mediterráneo; el grupo exclusivo de Cali no presentó cambios de aminoácidos no reportados.

Los cambios encontrados se relacionaron según las variantes del EBV (figura 4) y se evidenció que aquellos asociados con Raji presentaron la mayor cantidad de mutaciones, seguidos de los asociados con la variante Mediterráneo y la agrupación de Cali. Además, se observó que las mutaciones Ala210Asp (13 %) y Leu226His (27 %) eran exclusivas de los aislamientos relacionados con Raji. No se encontró ningún cambio exclusivo para los aislamientos relacionados con el Mediterráneo o con la agrupación de Cali.

## Discusión

En el presente estudio se reportan por primera vez los subtipos virales del EBV presentes en la cavidad oral de adolescentes de Cali, Colombia. El subtipo 1 se identificó en el 79 % las muestras positivas para EBV, en tanto que el subtipo 2 se identificó en el 19 %. Además, se reportó una exposición simultánea de los subtipos del 2 %.

Un mayor porcentaje del subtipo 1 del EBV era lo esperado, ya que es el subtipo predominante a nivel mundial, en tanto que el 2 es más frecuente en la región subsahariana de África.

En cuanto al porcentaje de homología del gen *EBNA3* del EBV, se ha reportado que el subtipo 1 difiere del subtipo 2 entre 10 y 20 %, y funcionalmente, el subtipo 1 ha demostrado gran capacidad para inmortalizar las células B en la infección primaria, en comparación con el subtipo 2 ^36^. La diferencia genómica global entre estos dos subtipos no se ha establecido del todo, puesto que se han secuenciado pocos genomas completos de subtipo 2 ^28^^,^^37^.

En los estudios de Chang, *et al*., que combinaron estudios poblacionales de subtipos en la población caucásica, se encontró que el 74 % estaba infectado con el EBV del subtipo 1, 19 % con el del subtipo 2 y el 7 % con ambos subtipos ^7^. Asimismo, en los estudios realizados en Argentina por Chabay, *et al*., se estableció que el subtipo del EBV predominante era el 1 ^38^. Por su parte, Correa, *et al*., reportaron la presencia del subtipo 1 en 75,9 % de pacientes argentinos adultos sanos seropositivos para el EBV, del subtipo 2 en el 17,6 % y en 6,5 % hubo infección simultánea con ambos subtipos ^39^.

En cuanto al bajo porcentaje de exposición simultánea en el presente trabajo, probablemente se debió a la poca edad de los participantes en el estudio. En los estudios realizados en China, Ai, *et al*., evaluaron 107 pacientes pediátricos con mononucleosis infecciosa y no se detectó la presencia simultánea de los dos subtipos de EBV en los niños, a pesar de haber padecido la enfermedad a temprana edad ^40^. En Argentina, Lorenzetti, *et al*., evaluaron 35 pacientes pediátricos con esta enfermedad y tampoco detectaron la presencia simultánea de los dos subtipos ^16^.

En los estudios recientes de análisis de los genomas completos del EBV, se ha establecido una gran variabilidad en las secuencias nucleotídicas del gen *BNLF1* que codifica para la proteína LMP1; estas variantes incluyen polimorfismos de nucleótidos únicos (*Single Nucleotide Polymorphisms*, SNP), deleciones, inserciones y un número variable de repeticiones en tándem ^30^^,^^41^. En este campo, los estudios se han enfocado en la presencia de variantes moleculares en la región C-ter de la proteína LMP1. Según Chang, *et al.*, la presencia de estas variantes podría alterar la señalización intracelular mediada por la proteína viral LMP1, por lo cual el análisis de variantes en esta región podría explicar el potencial patológico de la proteína ^7^.

En el presente estudio, la reconstrucción filogenética de las variantes de la LMP1 evidenció que ninguno de los EBV detectados en Cali se agrupó con la variante patogénica prototipo B95.8, predominante en población europea ^42^^,^^43^. Además, se observó que las variantes geográficas China 1, GD2, Cao y Akata se encuentran estrechamente relacionadas entre sí, al igual que las dos variantes geográficas del Mediterráneo, en tanto que las variantes geográficas China 2, NC y Alaskan conforman grupos independientes entre sí y con respecto a las otras variantes. Estos resultados concuerdan con los descritos por Edwards, *et al.*^26^*,* y más recientemente, por Saechan, *et al*. ^44^.

En un estudio realizado en Argentina en una cohorte de pacientes con mononucleosis infecciosa y linfomas positivos para EBV, en la secuenciación de la región C-ter de la proteína viral LMP1, la variante geográfica China 1 fue la de mayor frecuencia ^16^. Cuando los investigadores obtuvieron la secuencia completa del gen *BNLF1*, el análisis filogenético evidenció que aquellas variantes previamente clasificadas como China1 tenían una relación más cercana con la variante patogénica de la línea celular Raji ^45^, primera línea celular humana de origen hematopoyético obtenida en 1963 de un paciente nigeriano de 11 años con linfoma de Burkitt ^46^. En el presente trabajo, no se encontraron variantes de China 1 en los EBV presentes en Cali, sin embargo, se identificaron variantes patogénicas en la línea celular Raji.

Además, en los EBV detectados en Cali se identificaron variantes presentes en los aislamientos del EBV de origen en el Mediterráneo. En los estudios de Mei-Liao, *et al*., se determinó una distribución global de las variantes del Mediterráneo, las cuales se identificaron en poblaciones de países con geografías muy diferentes como Ghana, Brasil, Estados Unidos, Kenia, Argentina y Australia. Las variantes del Mediterráneo también se han encontrado en los EBV presentes en pacientes aparentemente sanos sin que se hayan relacionado directamente con enfermedades ^43^^,^^47^.

Un hallazgo importante del presente estudio fue que 13,7 % de los EBV en Cali presentaron variantes que no se identificaron ni agruparon con las variantes patogénicas de las líneas celulares o las variantes geográficas incluidas en el estudio. Se requerirán estudios adicionales para caracterizar dichas variantes y establecer si se relacionan con alguna enfermedad asociada con el EBV o si se trata de una variante geográfica propia de la ciudad de Cali.

Hasta el momento, los estudios de EBV en Colombia se han centrado en la relación del virus con el desarrollo de diferentes enfermedades. En Cali, Carrascal, *et al.,* examinaron 178 casos de carcinoma gástrico y detectaron el EBV en el 13 % de los pacientes, lo que sugiere una relación del virus con neoplasias malignas ^48^. Quijano, *et al*., detectaron el virus en el 56,7 % de personas con linfoma de Hodgkin ^9^. En pacientes pediátricos con trasplante hepático, Mesa, *et al.,* encontraron una relación entre el control de replicación del EBV y el grado de inmunosupresión de los pacientes ^49^.

El grado de variabilidad del gen *BNLF1* respalda la presencia de variantes moleculares observadas en los EBV aislados en Cali. Una posible explicación es que las proteínas de latencia del EBV, como la LMP1, podrían presentar una tasa de mutación en ciertas regiones de su estructura dependiente de la presión que ejerce el sistema inmunitario del huésped ^30^.

En un gran porcentaje (86,6 %) de las secuencias obtenidas de la región C-ter, se encontró el cambio de aminoácidos de glicina a serina en la posición 212 (Gly212Ser), cambio que se ha reportado con mucha frecuencia en varias partes del mundo ^50^^,^^51^, con excepción de Rusia, donde hasta el momento no se ha observado esta mutación ^52^ y sí, en cambio, la sustitución de un ácido glutámico a glutamina en la posición 328 (Glu328Gln) en pacientes y personas sanas, el cual no se ha reportado en otras regiones del mundo ^53^^,^^54^. En el presente análisis, no se encontró el cambio Glu328Gln, por lo que se validó la presencia de variantes del gen de la LMP1 únicas para ciertas regiones del mundo.

Una limitación del estudio fue el poco número de muestras del total disponible con EBV, pues se logró amplificar el producto de PCR de 576 pb de la región C-terminal de la LMP1 para la secuenciación en 29 de 84, debido probablemente a la fragmentación del ADN. Cabe anotar que, para la detección inicial del EBV, se amplificó un fragmento de 96 bp. Otra limitación fue el no encontrar estudios en que se hayan determinado subtipos y variantes del EBV en otras ciudades de Colombia, por lo que no fue posible hacer comparaciones.

En conclusión, en Cali está presente el subtipo 1 del EBV y las variantes Raji (patogénica) y Mediterráneo (geográfica); además, se detectó una variante que no se pudo agrupar con las variantes reportadas en la literatura especializada.
